# LKB1 Is an Essential Regulator of Spermatozoa Release during Spermiation in the Mammalian Testis

**DOI:** 10.1371/journal.pone.0028306

**Published:** 2011-12-01

**Authors:** Fiona C. Denison, Lee B. Smith, Phillip J. Muckett, Laura O'Hara, David Carling, Angela Woods

**Affiliations:** 1 Medical Research Council's Clinical Sciences Centre, Imperial College, London, United Kingdom; 2 Medical Research Council's Centre for Reproductive Health, University of Edinburgh, Edinburgh, United Kingdom; Clermont Université, France

## Abstract

LKB1 acts as a master upstream protein kinase regulating a number of kinases involved in diverse cellular functions. Recent studies have suggested a role for LKB1 in male fertility. Male mice with reduced total LKB1 expression, including the complete absence of the major splice variant in testis (LKB1_S_), are completely infertile. We sought to further characterise these mice and determine the mechanism underlying this infertility. This involved expression studies of LKB1 in developing germ cells, morphological analysis of mature spermatozoa and histological studies of both the testis and epididymis using light microscopy and transmission electron microscopy. We conclude that a defect in the release of mature spermatids from the seminiferous epithelium (spermiation) during spermatozoan development is a major cause of the infertility phenotype. We also present evidence that this is due, at least in part, to defects in the breakdown of the junctions, known as ectoplasmic specialisations, between the sertoli cells of the testis epithelium and the heads of the maturing spermatids. Overall this study uncovers a critical role for LKB1 in spermiation, a highly regulated, but poorly understood process vital for male fertility.

## Introduction

LKB1 is a serine/threonine protein kinase which has been implicated in a number of key cellular processes including the regulation of cell proliferation, cell polarity and energy metabolism [1]. Mutations in the human gene encoding LKB1 cause a rare disease called Peutz-Jeghers Syndrome [2]. Patients suffer many benign hamartomatous polyps of the gastrointestinal tract and display a predisposition towards malignant tumours [3]. In mice, global deletion of LKB1 is embryonic lethal; the embryos displaying defects in neural tube closure and vascular development [4].

LKB1 exists as a heterotrimeric complex in cells with the proteins STE20-related adaptor (STRAD) and mouse protein 25 (MO25). STRAD and MO25 have been shown to stabilise the LKB1 protein and greatly increase its catalytic activity. Their binding also causes LKB1 to relocalise from the nucleus to the cytoplasm [5], [6], [7], [8]. The emerging consensus view is that the LKB1 heterotrimer is constitutively active and that regulation occurs at the level of its downstream substrates or via changes in its localisation.

In 2003, LKB1 was shown to be a main upstream kinase responsible for the activation of AMP-activated protein kinase (AMPK) [7], [9], [10], a key regulator of cellular energy metabolism. Later studies implicated LKB1 in the regulation of 12 other kinases known as AMPK-related kinases [11], [12]. These are BRSK1 and BRSK2 (Brain-specific kinases 1 and 2); SIK1, SIK2 and SIK3 (Salt-inducible kinases 1–3); NUAK1 and NUAK2; MARK1-4 (Microtubule-affinity-regulating kinase 1–4) and SNRK (SNF1-related kinase). The role of some of the AMPK-related kinases is currently not well understood [13].

The *Lkb1* gene is composed of 10 exons, 9 of which are coding [2], [14], [15]. The human LKB1 protein has 433 amino acids (436 in mice). Amino acids 43–309 comprise the catalytic domain and this is flanked by N- and C- terminal domains, the functions of which are not well understood. Previously, we and others reported the existence of an alternative splice variant of LKB1 [8], [16], termed LKB1 short form (LKB1_S_) as opposed to the previously reported form of LKB1, termed LKB1 long form (LKB1_L_). The amino acid sequence of the two variants is identical apart from the C-terminus which is encoded by a different exon (exon IXa in the case of LKB1_S_ or IXb in the case of LKB1_L_). Both splice variants show a similar sub-cellular localisation and comparable catalytic activity towards AMPK and a number of AMPK-related kinases [8], [16], [17]. We reported previously that the LKB1_S_ protein is primarily expressed in the testes. Western blotting of total cell extracts from testis show that LKB1_S_ is the most abundant LKB1 splice variant in this tissue, although some LKB1_L_ is also detectable [8]. LKB1_S_ is also present in human testis [16].

The testis is a highly specialised organ and contains many cell types necessary for the highly coordinated process of spermatogenesis, the mechanism by which undifferentiated diploid spermatogonia develop into haploid spermatozoa. Spermatogenesis can be divided into four main phases; proliferation, meiosis, spermiogenesis and spermiation which progress within the seminiferous epithelium, from the outside of the seminiferous tubule toward the lumen. Sertoli cells present within the epithelium play a major role in supporting germ cell development and sertoli-germ cell junctions are thought to allow for continuous communication between these cell types. As spermatogenesis occurs in waves along the seminiferous tubules, the stage of spermatogenesis will vary at different lengths along the tubule and can be assigned a number (I-XII in mice). Following development within the seminiferous tubules, the spermatids are released into the lumen and from here move into the epididymis where further maturation occurs. The epididymis is divided into three sections, the caput epididymis (upper section), corpus epididymis (central section) and cauda epididymis (lower section). It is in the cauda epididymis that the sperm are stored prior to release [18].

In a recent independent study, male mice displaying significantly reduced total LKB1 expression were shown to be completely infertile, the only overt phenotypic abnormality detected [16], [19]. In addition to possessing lower levels of LKB1_L_ protein in most tissues, these mice completely lack expression of the LKB1_S_ splice variant, the primary form of LKB1 in the testis. This has led to the hypothesis that the LKB1_S_ splice variant is involved in processes important for male fertility. In this study, we have further characterised these mice, which we term *LKB1_S_KO*, to establish the intra-testicular mechanisms underlying the observed infertility phenotype. During the final phase of spermatogenesis, termed spermiation, mature spermatids become detached from the supporting sertoli cells and are released into the lumen of the seminiferous tubule (see [20] for a recent review). Here we show that spermiation is defective in the absence of LKB1_S_.

## Materials and Methods

### Antibodies

Mouse anti-LKB1 monoclonal antibody (Ley37 D/G6), which recognizes both splice variants of LKB1, was from Santa Cruz Biotechnology. Anti-tubulin Yol1/34 antibody and rabbit anti-MARK3 was from Abcam. Mouse monoclonal antibody recognising the spermatozoan acrosome (Mab 18.6) was a kind gift from Prof. Harry Moore (University of Sheffield, UK). Anti-NUAK2 raised against residues 653–673 of human NUAK2, anti-BRSK2 raised against a C-terminal peptide sequence of the human protein, and anti-SNRK antibody raised to residues 737–753 of human SNRK were kind gifts from Prof. Dario Alessi (University of Dundee, UK). Phospho-SIK1 antibody was raised in rabbit against the peptide, CKSGEPLS(pT)WCGSPPY. SIK2 antiserum was a kind gift from Prof. Hiroshi Takemori (Osaka, Japan). Antibodies recognizing AMPKα1 and AMPKα2 were a kind gift from Prof. Grahame Hardie (University of Dundee, UK) [21]. Secondary antibodies conjugated to Alexa-Fluor 488/568/680 or IRDye800 were purchased from Invitrogen and Li-COR respectively.

### Animals

All procedures were in accordance with the UK Home Office Animal Procedures Act of 1986 under licence number PPL 70/6670. Approval was also given by the Imperial College Animal Ethics Committee. Production of mice harbouring *Lkb1* floxed alleles (*LKB1_S_KO*) has been described previously [19], [22].

### Tissue harvesting and preparation

Male mice (C57/Bl6) at approximately 12 weeks were culled by cervical dislocation. Tissues were harvested and immediately frozen in liquid nitrogen. Prior to analysis, tissues were roughly chopped in two volumes of ice-cold buffer A and briefly homogenized with a rotor-stator homogenizer. 1% (v/v) Triton X-100 was added and the homogenates were incubated on ice for 10 min, sonicated in a 4°C water bath for 3 cycles of 20 sec, and then centrifuged at 16,000 x g for 15 min to remove insoluble material. Protein concentration in cell and tissue lysates was determined using the Bradford assay [23].

### Western blot analysis

All tissue homogenates for blotting LKB1 were first pre-cleared with protein A-Sepharose to remove IgG. Samples were resolved on 10–12% polyacrylamide gels by SDS-PAGE. Primary antibodies were detected using secondary antibody conjugated to either Alexa-Fluor 680 or IRDye800 and scanned on the Li-COR Odyssey Infrared Imaging System. Quantification of results was performed using Odyssey software 2.0 (LI-COR Biotechnology).

### Mating assays

Males: 8 wild-type and 8 *LKB1_S_KO* mice (8–20 weeks old) were paired with at least two females each for up to 14 days. Females were checked for vaginal mucus plugs for 2 days and pregnancies noted after the appropriate time.

### Sperm counts

Cauda epididymides were excised and placed in 1 ml M2 medium (Sigma) (both epididymides per mouse). Several incisions were made and the spermatozoa allowed to disperse into the medium for 15 minutes at room temperature. Sperm were counted on a haemocytometer and the mean number of spermatozoa collected per mouse calculated.

Cells were only included in the counts if they could be recognised as a spermatozoan at low magnification, i.e., a head with a flagellum attached, regardless of whether they looked morphologically abnormal. Detached heads and tails and round cells were not included.

### Spermatozoa immunofluorescence

Mouse cauda epididymides were excised and placed in 0.5 ml M2 medium (Sigma). Several incisions were made and the spermatozoa allowed to disperse into the medium for 15 minutes at room temperature. Aliquots of the suspension were pipetted onto polylysine coated glass slides, air dried and fixed in methanol. Acrosomes were stained with Mab 18.6 monoclonal antibody, which recognises a specific antigen on the acrosomal surface [24], and tails were stained with an anti-tubulin antibody. Primary antibodies were detected with Alexa fluor linked secondary antibodies. Nuclei were stained with DAPI. Slides were viewed by sequential scanning of each wavelength on a Leica TCS SP1 confocal microscope and analysed with Leica software.

### Tissue preparation for light microscopy (LM) and transmission electron microscopy (TEM)

After glutaraldehyde fixation and processing, samples were embedded in araldite. Semi thin sections of 0.5–1 µm were stained with toluidine blue in borax. Ultra-thin sections were stained in uranyl acetate followed by Reynold's lead citrate.

### Quantification of seminiferous epithelium thickness

Seminiferous epithelium thickness was measured in testis thin-sections from six LKB1_S_KO and five wild-type mice by subtracting the seminiferous tubule radius from the tubule lumen radius of at least 100 tubules per section, as previously described [25].

### Immunoprecipitation and Activity Assays

All tissue homogenates were first pre-cleared with protein A or G-Sepharose. AMPK, AMPK-related kinases or LKB1 complexes were immunoprecipitated from soluble tissue homogenates using antibodies bound to protein A/G-Sepharose. After extensive washing, kinase activity present in the immune complexes was determined as previously described [22], [26].

### Quantitative RT-PCR analysis

RNA was isolated from testis (n = 5–7 testes per age) by homogenization in Trizol reagent (Invitrogen) according to the manufacturer's instructions, followed by purification on an RNeasy column (Qiagen). 2 µg RNA was used for first strand cDNA synthesis using Superscript II (Invitrogen) according to the manufacturer's instructions. For LKB1_L_ and total LKB1, quantitative PCR was performed with SensiMix Plus SYBR kit (Quantace) using Opticon DNA Engine. Total LKB1 was amplified using primers spanning exons 2 and 3 (forward: ggacgtgctgtacaatgagg, reverse: gcatgccacatacgcagt). Primers spanning exons 9 b and 10 were used to specifically amplify LKB1_L_ (forward: cctgcaagcagcagtgac, reverse: ccaacgtcccgaagtgag). Detection of transcripts was done using Roche Universal Probe Library (Roche, Welwyn, UK). For LKB1_S_, transcripts were amplified using primers spanning exons 8 and 9a (forward: cattatctacacccaggacttcaca, reverse: cgcatgcatcctcgctaa). The LKB1_S_ specific product was detected using Fam labelled probe: aggaggcggccgag (from Integrated Technologies Inc). ABI Prism 7500 Sequence Detection System (Applied Biosystems) was used for detection according to the manufacturer's instructions. Expression of all samples was compared to that of GAPDH.

### Immunohistochemistry

Single colorimetric immunohistochemistry on Bouin's-fixed testicular tissue was performed as previously described [27] (n = 5 testes per age).

## Results

### Male mice lacking LKB1_S_ protein are infertile

**Figure 1:**
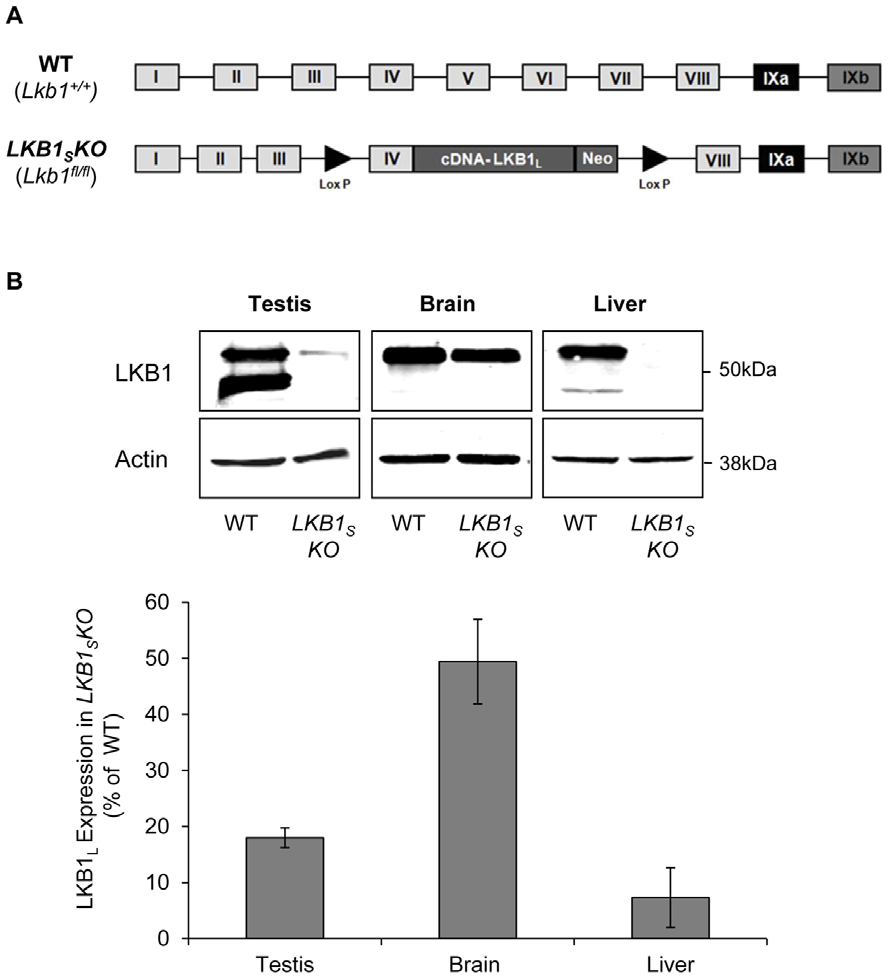
LKB1 expression in *LKB1_S_KO* mice. **A**) Generation of *LKB1_S_KO* mice, adapted from [19]. Exons IV–VII (encoding the kinase domain) of the wild-type (WT) *Lkb1* gene were replaced by a cassette encompassing exon IV plus cDNA encoding the rest of LKB1_L_. This cassette was flanked by lox-P excision sites (▸) and contained a neomycin (Neo) gene for selection. Mice homozygous for expression of this cassette (*Lkb1^fl/fl^*) no longer express LKB1_S_ and are *de facto LKB1_S_KO* mice. **B**) Mouse tissues homogenates were analysed by western blotting with a monoclonal antibody raised against total LKB1. An anti-actin antibody was used to show equal loading. Representative blots are shown and the migration of molecular mass standards are as indicated. **C**) Quantification of the relative intensity of the LKB1_L_ band between wild-type and *LKB1_S_KO* mice is shown in the bar graph below. Results are plotted as the percentage expression of LKB1_L_ in *LKB1_S_KO* mice compared to wild-type and are the means±S.E.M. of three separate blots from three individual mice.

*LKB1_S_KO* mice were originally generated during the development of a conditional allele of LKB1 [19] (Figure 1A). *LKB1_S_KO* mice are homozygous for the presence of a ‘floxed’ *Lkb1* allele (*Lkb1^fl/fl^*). Western blot analysis of testis, brain and liver extracts, using a monoclonal antibody that recognises both splice forms of LKB1, shows that LKB1_S_ is undetectable in tissues isolated from *LKB1_S_KO* mice confirming functional deletion of LKB1_S_. The expression of LKB1_L_ is also dramatically reduced (>80% in testis, >50% in brain and >90% in liver) confirming the previously reported effect of the floxed allele on LKB1_L_ expression [19], [22] (Figure 1B).

In order to confirm the male infertility previously reported [19], mating assays were conducted. Males were paired with at least two females each for up to 14 days, and mating determined by the presence of copulation plugs in the mated females. Plug checks were discontinued once every male had plugged at least one female. This indicated that although *LKB1_S_KO* mice did attempt to mate, no pregnancies were observed in the females paired with *LKB1_S_KO* males, whereas around 70% of females paired with wild-type mice became pregnant (Table 1). No fertility problems were noted in female *LKB1_S_KO* mice as the ratio of pregnancies of *LKB1_S_KO* females paired with wild-type males was the same as that shown by purely wild type crosses (75%) over the breeding programme of more than 30 pairings.

**Table 1:** Mating Assays.

Male Genotype	Number of males	Number of females paired	Numberof males that plugged females	Pregnancies
WT	8	26	8	18
*LKB1_S_KO*	8	26	8	0

### Mature spermatozoa are absent in the epididymis of *LKB1_S_KO* mice

In order to elucidate the infertility of *LKB1_S_KO* mice, spermatozoa were collected from the cauda epididymides, where mature spermatozoa are stored, and sperm count calculated. An average of 2.7×10^7^ spermatozoa were collected from each wild-type mouse, whereas only 2.8×10^5^ spermatozoa were collected from each *LKB1_S_KO* mouse, a reduction of >95%. There was no significant difference in sperm counts between wild-type mice and mice heterozygous for the floxed allele (*Lkb1^+/fl^*).

**Figure 2:**
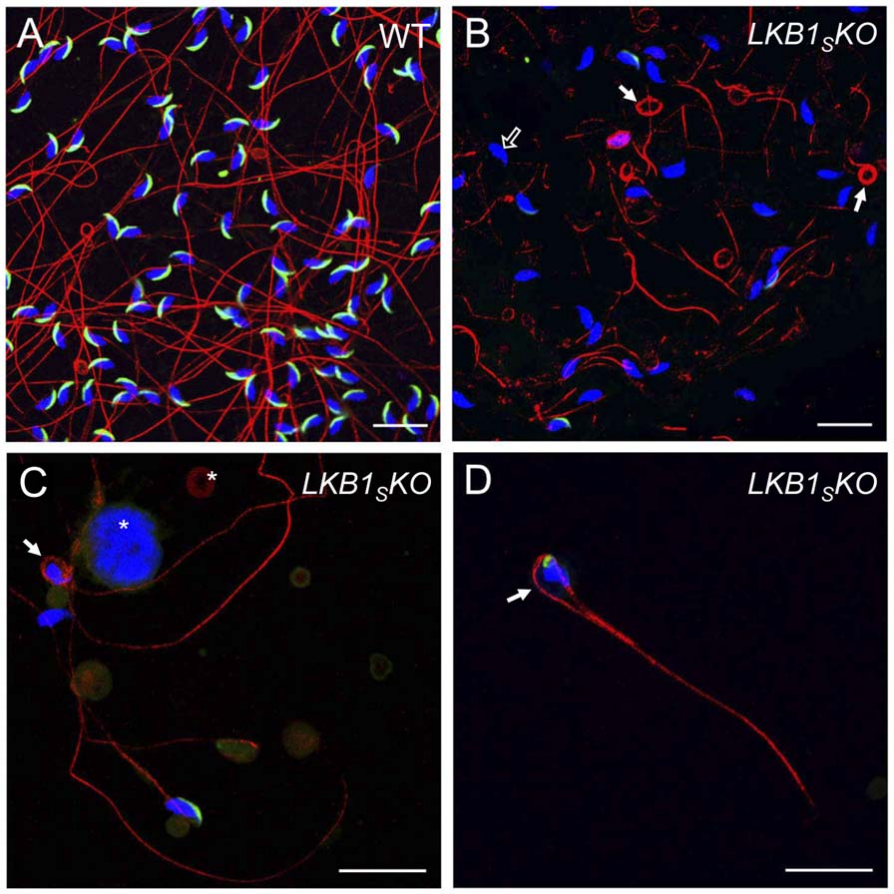
Fluorescence microscopy images of mature spermatozoa. Spermatozoa were taken from the cauda epididymis of wild-type (**A**) and *LKB1_S_KO* mice (**B**–**D**), and visualised by immunofluorescence. Tails were stained with an anti-tubulin antibody (red), acrosomes with an anti-acrosomal monoclonal antibody (green) and nuclei with DAPI (blue). Sperm from LKB1_S_KO mice frequently show coiled tails (*filled white arrows*). In (**D**), the tail has formed a ‘lasso’-type structure around an abnormally shaped nucleus. Sperm nuclei from LKB1_S_KO mice often lack acrososmes (*open white arrow* in **B**) as shown by the lack of green fluorescence at the anterior nuclear surface. Abnormal cellular debris is visible in LKB1_S_KO samples, as indicated by *asterisks* in (**C**). Slides were viewed on a Leica TCS SP1 confocal microscope. Images are representative of at least three mice (Scale bar  = 20 µM).

Spermatozoan morphology from the *LKB1_S_KO* mice was analysed by fluorescence microscopy (Figure 2). An anti-tubulin antibody was used to stain the flagellum and DAPI used to stain the nucleus. In addition, the slides were incubated with the Mab18.6 antibody that recognises an antigen within the spermatozoan acrosome. The acrosome is a secretory organelle containing digestive enzymes which are released upon binding to the ovum [28]. Spermatozoa from a wild-type mouse are shown in Figure 2A. The nucleus is falciform in shape and the acrosome appears as a crescent-shaped structure extending over the anterior nuclear surface. Spermatozoa from *LKB1_S_KO* mice display a number of abnormalities. Heads and tails are often separated and many tails appear fragmented (Figure 2B). Tails are frequently coiled, sometimes around the head (Figure 2 B,C), and sometimes appearing as a ‘lasso’-type structure (Figure 2D). Approximately 70% of spermatozoa heads from *LKB1_S_KO* mice have no detectable acrosome staining at all, many of the remainder displaying an abnormal staining pattern, with the acrosomes often being reduced in size. Only 10–15% of spermatozoa from wild-type mice have no acrosomal staining. Overall, more cellular debris is visible amongst the spermatozoa from *LKB1_S_KO* mice (Figure 2C). There were no differences apparent between the morphology of spermatozoa from wild-type mice and mice heterozygous for the floxed allele (*Lkb1^+/fl^*) (data not shown).

**Figure 3:**
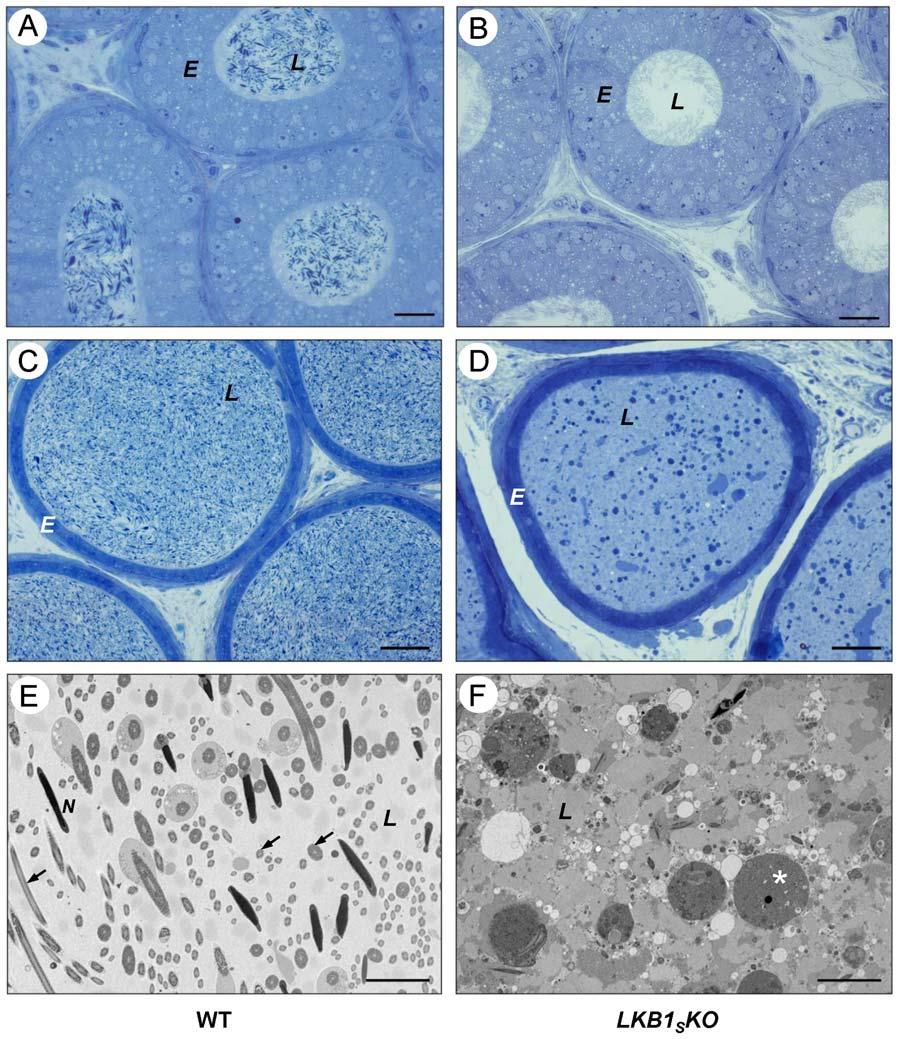
Histology of the cauda and caput epididymis. Cauda epididymis (**A**–**B**) and caput epididymis (**C**–**D**) sections were visualised by light microscopy. Representative images are shown from observations of three mice per genotype. Epididymal epithelia *(E)* and tubule lumens *(L)* are labelled. (**A**) and (**C**) show sections through wild-type epididymis showing an abundance of spermatozoa within the lumen. (**B**) and (**D**) show sections through the epididymis of *LKB1_S_KO* mice showing abnormal structures within the lumen and very few spermatozoa. (**E**–**F**) TEM images of the cauda epididymal lumen from a wild-type mouse (**E**) and a *LKB1_S_KO* mouse (**F**). The wild-type section shows numerous cross-sections through sperm heads (*N*) and tails (*arrows*). The section from a *LKB1_S_KO* mouse shows dense luminal fluid (as indicated by the darker background to WT), cellular debris, abnormal round structures (*asterisk*) and an absence of recognisable spermatozoa cross sections (A and B, scale bar  = 20 µm; C and D, scale bar  = 50 µm; E and F, scale bar  = 5 µm).

Light microscopic (LM) and transmission electron microscopic (TEM) analysis of sections of cauda and caput epididymis shows dramatic differences in the contents of the lumen between wild-type and *LKB1_S_KO*. In wild-type sections (Figure 3 A,C,E), the lumen has an abundance of spermatozoa, as recognised by deeply staining nuclei, whereas in the *LKB1_S_KO* mice (Figure 3 B,D,F), there are very few identifiable spermatozoa. In the caput epididymis the majority of lumens appear empty in *LKB1_S_KO* animals. However, the cauda epididymis is filled with abnormal cellular debris including numerous deeply staining round structures of various sizes, most likely degrading germ cells. No obvious differences in the structure of the epididymal epithelium were apparent between *LKB1_S_KO* and control animals.

### Histological abnormalities in testis from *LKB1_S_KO* mice

**Figure 4:**
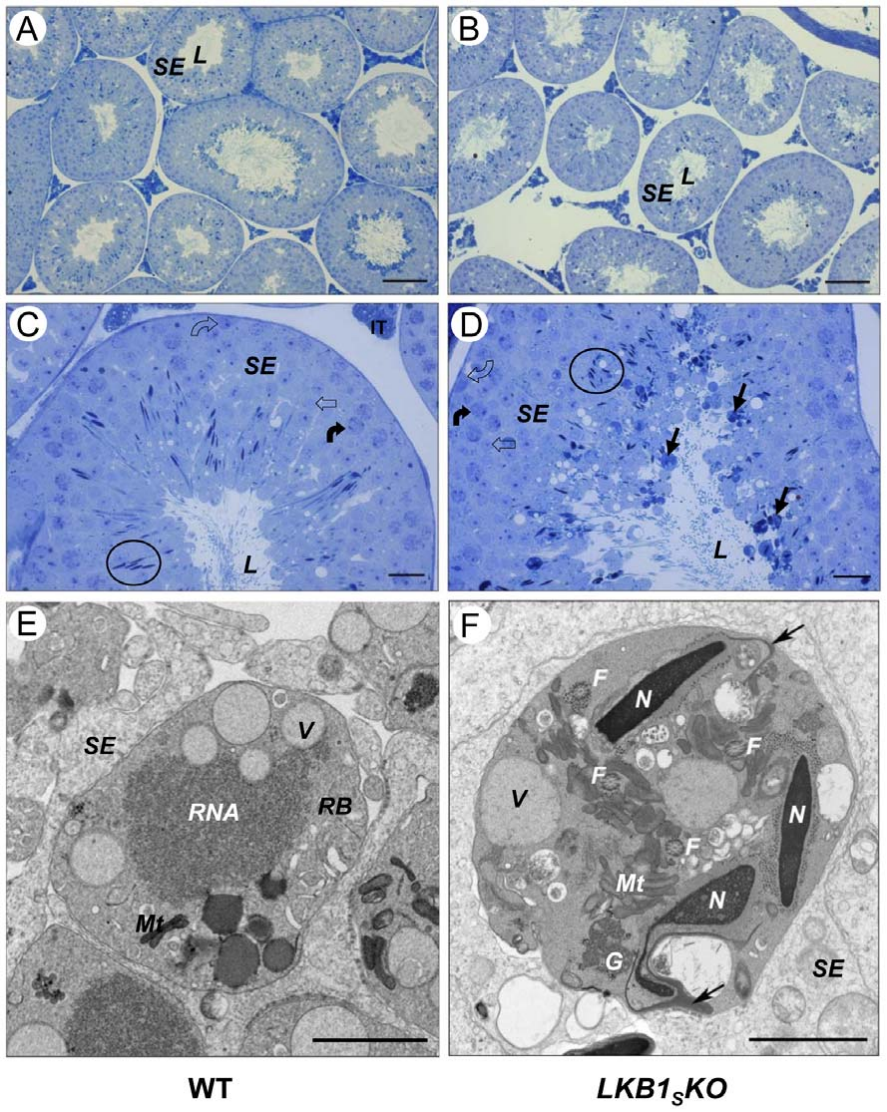
Testis histology. Cross-sections of seminiferous tubules from wild-type (left-hand panel, **A and C**) and *LKB1_S_KO* (right-hand panel, **B and D**) mice. The seminiferous epithelium (*SE*), which is made up of sertoli cells and developing germ cells, is indicated. This surrounds the lumen (*L*) of the tubules. (**C**) and (**D**) show sections from tubules at approximately stage V. Circles have been used to show the nuclei of elongating spermatids. Spermatogonia (*open curved arrow*), spermatocytes (*closed curved arrow*) and round spermatids (*open arrow*) are also indicated. A number of dense, round bodies of varying sizes can be seen around the lumens from *LKB1_S_KO* mice. Examples of these are indicated in (**D**) (*closed black arrows*). Representative images are shown (A and B, scale bar  = 100 µm; C and D, scale bar  = 20 µm). (**E**) and (**F**) show TEM images of a residual body *(RB)* within the seminiferous epithelium *(SE)* from a wild-type mouse (**E**), and an abnormal cytoplasmic body from a LKB1_S_KO mouse (**F**). Normal residual often contain such structures as vacuoles (*V*), RNA and mitochondria (*Mt*). The abnormal cytoplasmic bodies seen in (**F**) are similar to residual bodies but often contain at least one condensed spermatid nuclei *(N)* and several cross sections of flagella *(F)* as indicated. Detached acrosomes, identified as the deeply staining crescent shape structures close to the anterior nuclear surface, are indicated with an arrow. Granular material is also indicated *(G)*, (E and F, scale bar  = 2 µm).

To establish the reason for the large reduction in numbers of correctly developed spermatozoa in the epididymis, testes were harvested from age-matched wild-type and *LKB1_S_KO* mice. There was no significant difference in testis weight between the genotypes (data not shown). Analysis of testis sections by LM showed no differences in the thickness of the seminiferous epithelium between wild-type (73±2.9 µm, n = 5) and *LKB1_S_KO* (77±2.2 µm, n = 6) mice (Figure 4 A,B).

Examination of tubule structure in LM and TEM images did not show any major differences in the appearance of spermatogonia, spermatocytes and round spermatids. However, the luminal interface of the seminiferous epithelium appeared disorganised in *LKB1_S_KO* testes compared to wild-type. In addition, there were numerous, often large, darkly staining bodies present at the luminal interface of *LKB1_S_KO* tubules (Figure 4 B,D). These dense bodies were analysed at higher magnification by TEM. This showed them to be similar in appearance to residual bodies that are formed during normal spermatogenesis in wild-type mice (Figure 4E). Residual bodies are formed at spermiation when the excess cytoplasm (cytoplasmic lobe) from adjacent spermatids fuses and condenses. They often contain vacuoles, RNA and organelles such as mitochondria. In LKB1_S_KO mice, unlike normal residual bodies, these bodies frequently contain at least one condensed spermatid nucleus along with flagella (Figure 4F). Dense granular material is also visible, whilst acrosomes are often completely detached from the spermatid nucleus, frequently remaining attached to the sertoli cells at the cell periphery, with the detached nucleus left surrounded by cytoplasm.

### Defective spermiation in *LKB1_S_KO* mice

**Figure 5:**
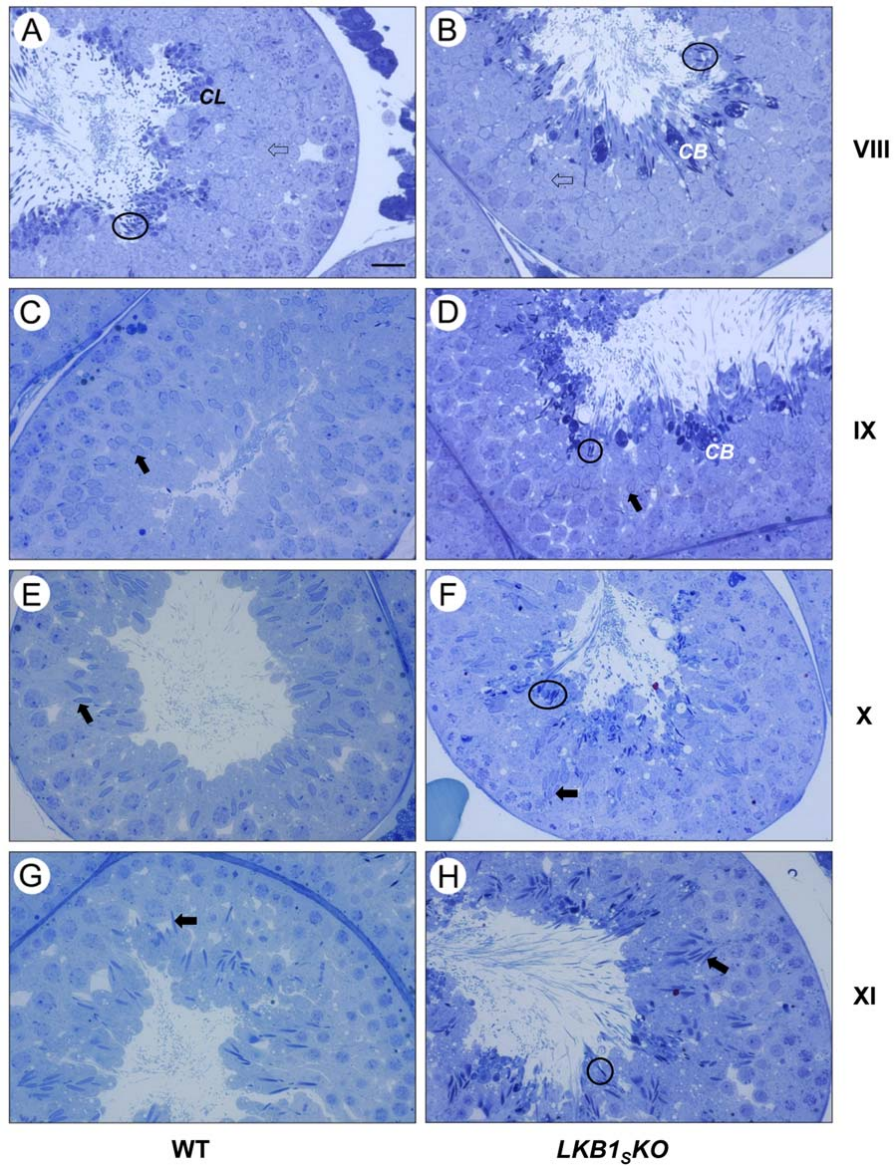
LM images showing ‘failure of spermiation’ in *LKB1_S_KO* mice. Representative images of seminiferous tubules are shown at stage VIII (**A,B**), when spermiation normally occurs; and stages IX, X, and XI (**C**–**H**), after spermiation has normally taken place. The stage numbers are shown to the right of the images. Elongated spermatids are identifiable by their darkly-staining, condensed nuclei. The nuclei of immature round spermatids (*open arrows*) and elongating spermatids (*closed arrows*) are less deeply stained and can be seen embedded within the epithelium at the relevant stages of both WT and LKB1_S_KO sections. There is a progressive condensation and elongation of the nucleus of the elongating spermatids from stage IX to stage XI. Sections from wild-type mice are displayed on the left. At stage VIII, elongated spermatids (*examples circled*) and cytoplasmic lobes *(CL)* are visible around the lumen. There are no elongated spermatids present around the lumen after stage VIII in wild-type sections. In contrast, in tubules from *LKB1_S_KO* mice, shown on the right, elongated spermatids are visible around the lumen at all stages displayed (*examples circled*). In addition, abnormal deeply staining cytoplasmic bodies *(CB)* can be seen around the lumen, (scale bar  = 20 µm).

Condensed elongated spermatids are released into the lumen of the seminiferous tubules at stage VIII of spermatogenesis in a process known as spermiation [29]. In wild-type testes, at stage VIII the elongated spermatids are still visible attached to the sertoli cells at the luminal interface. The cytoplasmic lobes can be recognised, as the more deeply staining cytoplasm now positioned basally to the spermatid heads (Figure 5A). By stage IX, the condensed spermatids have been released and most of the residual bodies have been phagocytosed. In contrast, elongated spermatids are retained through stages VIII, IX, X, and XI in the tubules from *LKB1_S_KO* animals, showing a failure of spermiation (Figure 5 B,D,F,H). There are notably fewer by stage XI suggesting many of the elongated spermatids have either been released or phagocytosed by the sertoli cells by this stage (Figure 5 B,D,F,H). Overall, the majority of elongated spermatids were retained such that they were present in all seminiferous tubules analysed from *LKB1_S_KO* mice, whereas they were only present in stage I–VIII tubules from wild-type mice (data not shown).

Overall, no obvious differences were detected in the number of elongated spermatids visible in stage I to VIII tubules between wild-type and *LKB1_S_KO* seminiferous epithelium, or the numbers of round and elongating spermatids present in the appropriate stages.

**Figure 6:**
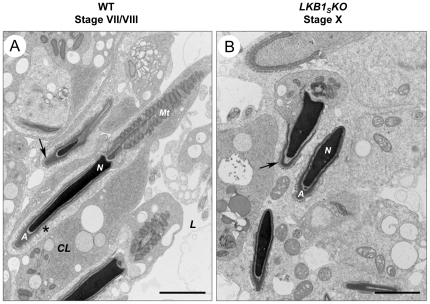
TEM images of cell junctions between sertoli cells and spermatids. **A)** Elongated spermatids are shown around the lumen from a wild-type stage VII tubule before spermiation. The residual spermatid cytoplasm can be seen around the spermatid nucleus *(N)* as a cytoplasmic lobe *(CL)*. Although the actin bundles of ectoplasmic specialisations are still visible around some spermatids (*arrow*), they are beginning to break down around others as indicated by an *asterisk*. Mitochondria aligned along the spermatozoan flagellum are labelled *(Mt)*. Spermatid acrosomes *(A)* and the tubule lumen *(L)* are indicated. **B)** Elongated spermatids are shown around the lumen from a stage X *LKB1_S_KO* tubule. These spermatids would normally have been released at stage VIII. Areas of ectoplasmic specialisations are still visible around the retained spermatid heads as indicated by an *arrow*, (scale bar  = 2 µm).

For spermiation to proceed correctly a series of highly regulated steps is required. One of the key processes required for spermiation to occur is the breakdown of junctions between spermatid heads and sertoli cells, known as ectoplasmic specialisations [29]. These ectoplasmic specialisations can be recognised at the EM level by the presence of deeply staining actin bundles around the spermatid heads. The regulation of these junctions is not well understood [30]. Analysis of the structure of ectoplasmic specialisations by TEM shows that they appear normal in *LKB1_S_KO* mice. However, in *LKB1_S_KO* mice it appears that they are not breaking down at the correct stage. At stage VII/VIII, just prior to spermiation in wild-type tubules, the excess cytoplasm has formed a ‘hood’ over the nucleus and the ectoplasmic specialisations are beginning to break down (Figure 6A). Conversely, in stage X tubules from *LKB1_S_KO* testes partial regions of the actin bundles from ectoplasmic specialisations can still be seen adjacent to the mature spermatid heads (Figure 6B). This suggests that a defect in the breakdown of ectoplasmic specialisations is playing a role in the failure of spermiation.

### LKB1 is localised in the cytoplasm of meiotic and post-meiotic germ cells

**Figure 7:**
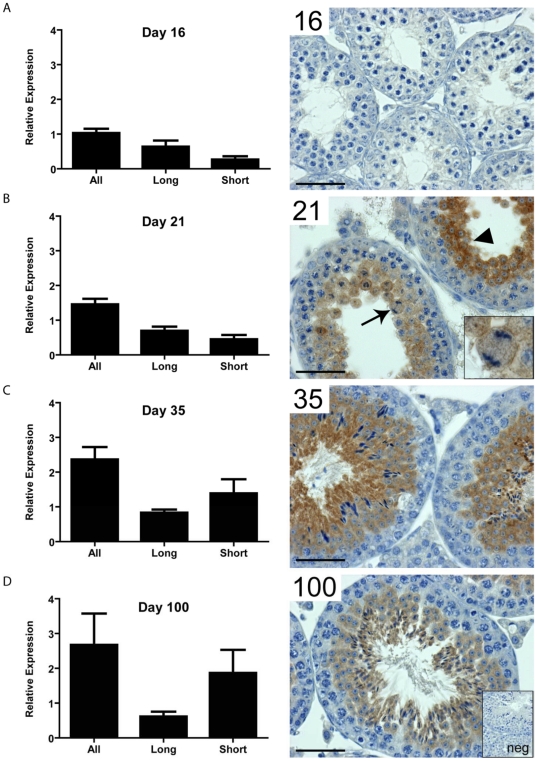
LKB1 splice variant relative gene expression and testicular localisation. **A)** At day 16, prior to meiosis, testicular LKB1 is expressed at relatively low levels, with LKB1 protein below the detectable limits of colourimetric immunohistochemistry. **B**) At day 21 total LKB1 expression has increased, predominantly through increased expression of LKB1_S_. LKB1 protein can be detected in meiotic spermatocytes (inset and arrow), and post-meiotic spermatids (arrowhead). At day 35 (**C**) and day 100 (**D**), the predominant transcript is LKB1_S_ with LKB1 protein localised to the cytoplasm of elongated spermatids in addition to round spermatids and spermatocytes, (scale bar  = 50 µm). The insert labelled ‘neg’ shows a negative control in which no primary antibody was incubated with the tissue.

To determine the normal expression pattern of LKB1 in the developing postnatal testis and the significance of the LKB1_S_ transcript, expression of the two splice forms was determined. During testis development in young mice, the first wave of spermatogenesis occurs synchronously across all seminiferous tubules so that all tubules are at an equivalent point in the cycle. Different germ cell generations appear in mice of specific ages (primary spermatocytes day 14, round spermatids day 23, elongate spermatids, day 32) [31] and so changes in gene expression over time can reflect changes in the germ cell population providing an insight into cell-specific expression. The expression of the two LKB1 splice variants was analysed by RT-PCR in wild-type testis from mice at age 16, 21, 35 and 100 days. Total LKB1 transcript increases almost 3-fold throughout this developmental period. This increase was primarily accounted for by an increase in LKB1_S_. Whereas the expression of LKB1_L_ does not greatly alter between the four time points, the expression of LKB1_S_ increases about 6-fold from day 16 to day 100, suggesting high expression of LKB1_S_ in later generations of germ cells (post-meiotic). Indeed, immunohistochemistry using an antibody specific to total LKB1, showed minimal expression in testis from 16 day old mice, but confirmed expression from day 21 onwards in meiotic and post-meiotic germ cells (Figure 7).

### Analysis of Potential Downstream Substrates for LKB1 in Testis

**Figure 8:**
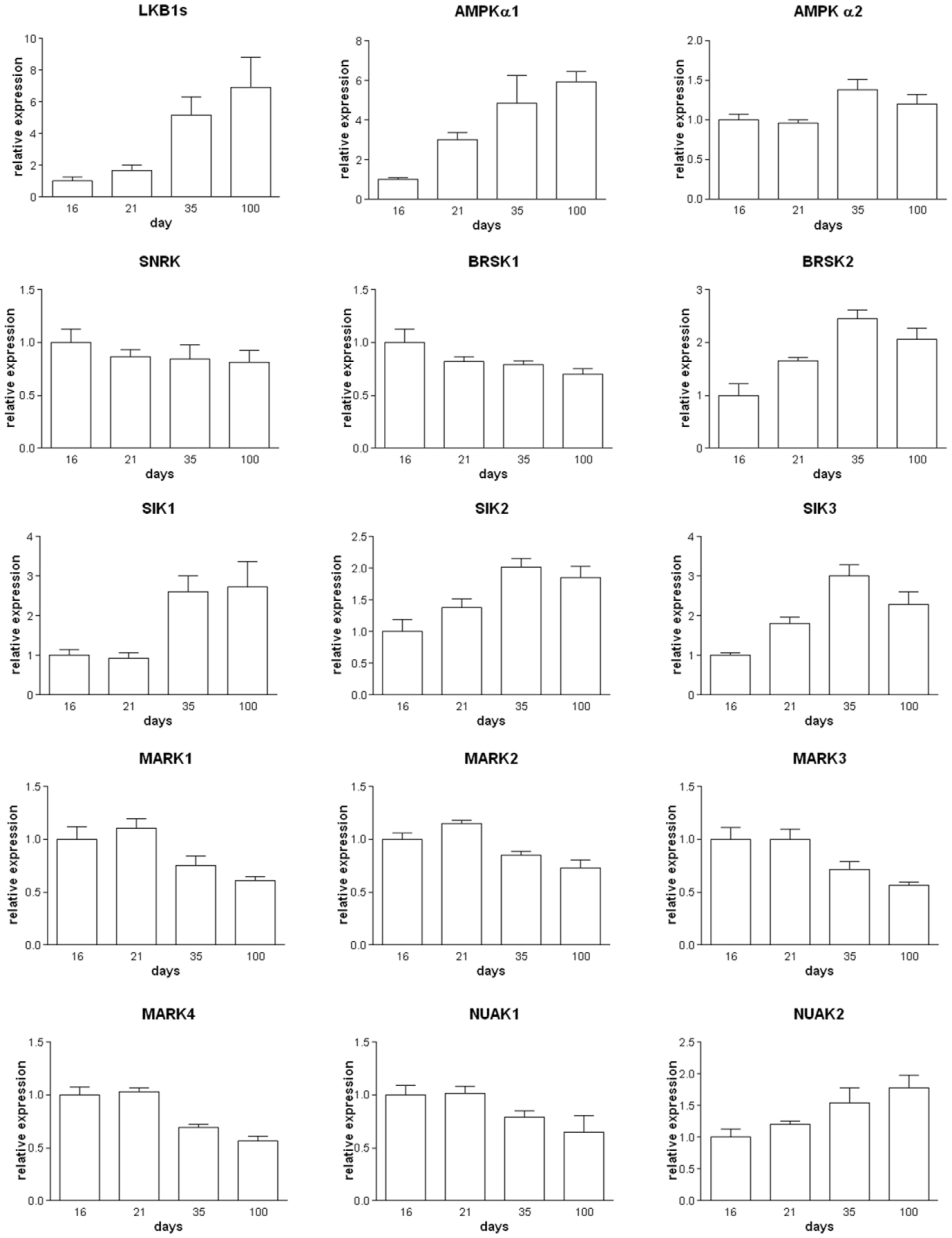
Expression of AMPK and AMPK-related kinases in testis. The mRNA expression levels of LKB1 and downstream kinases in developing testis at post-partum days 16–100. Values are shown relative to the expression levels at day 16 and shown as the mean +/- SEM, n = 5.

In order to determine which downstream targets of LKB1 may be responsible for the failure of spermatid release in the *LKB1_S_KO* tubules we analysed the RNA expression levels of AMPK and AMPK-related kinases in testes of developing mice. We reasoned that downstream targets of LKB1 in the testis are likely to have similar expression patterns to that of LKB1_S_ (Figure 8). The expression of several of the RNAs examined followed a similar pattern through development to that of LKB1_S_. AMPKα1 and α2, SIK1, SIK2, SIK3 and BRSK2 all show large, significant increases in expression between day 21 and 35, similar to that of LKB1, making them possible candidates for a role downstream of LKB1 in male spermiation. Interestingly, SNRK which has previously been reported to have testis specific expression in rats [12], does not have a similar RNA expression pattern to LKB1_S_ but rather has unaltered expression through 16–100 days suggesting it is not expressed at significant levels in postmeiotic germ cells. The expression levels of AMPK or AMPK-related kinase mRNAs were not significantly different between wild type and *LKB1_S_KO* testis between 21 and 35 days (data not shown). This suggests that these downstream targets of LKB1 are not under transcriptional regulation by LKB1. Regulation of the downstream targets of LKB1 is likely to involve post-transcriptional mechanisms, for example, phosphorylation.

**Figure 9:**
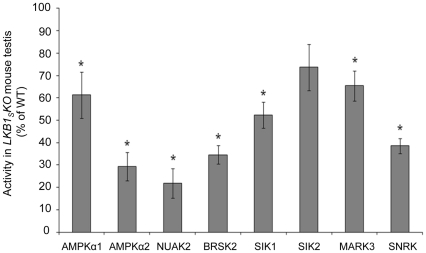
Activity of AMPK and AMPK-related kinases in *LKB1_S_KO* testis. Activity measured in immune-complexes using antibodies specific for AMPK and AMPK-related proteins are plotted as a percentage of the activity measured in wild type testis and shown as the mean±S.E.M. from three individual mice. * indicates a statistically significant difference in activity in *LKB1_S_KO* compared to wild-type samples (*p*<0.05 with Student's unpaired t test).

In order to further investigate possible downstream substrates of LKB1_S_ in testis, the activity of AMPK and various AMPK-related kinases was compared in testis tissue extracts from wild-type and *LKB1_S_KO* mice. Antibodies raised against AMPKα1, AMPKα2, NUAK2, BRSK2, SIK1, SIK2, MARK3 and SNRK were used to immunoprecipitate the proteins from testis homogenates and activity in the immune complexes was determined using the AMARA peptide assay. The activity of other AMPK-related kinases was not determined due to a lack of suitable antibodies. Of the eight kinases measured, the activities of AMPKα2, NUAK2, BRSK2 and SNRK were most reduced in *LKB1_S_KO* mice compared to wild-type (by approximately 60–75%) (Figure 9).

## Discussion

In this study, we describe the infertility phenotype of male mice which display significantly reduced LKB1 expression. These mice show a complete absence of a testis-specific LKB1 splice variant, LKB1_S_. Our study confirms and extends the results of a previous study that reported male infertility in *LKB1_S_KO* mice [16]. Importantly, our study identifies a defect in the release of mature spermatids from the seminiferous epithelium of the testis (spermiation) as a major cause of the observed infertility phenotype

There is almost a complete absence of mature spermatozoa in the cauda epididymis of *LKB1_S_KO* mice. The few spermatozoa present are immotile and abnormal in terms of morphology, often showing coiled and fragmented tails, absence of an acrosome and abnormal head shapes. Surprisingly, testis weight, which can give an indication of the number of germ cells present and if there is a severe disruption of spermatogenesis [18], was unaltered in the *LKB1_S_KO* mice. In addition, analysis of testis histology did not show any obvious reductions in different germ cell types.

The release of mature spermatozoa into the lumen of the seminiferous tubules occurs at stage VIII of spermatogenesis [29]. Analysis of tubules at different stages of the spermatogenic cycle indicated that spermiation was not occurring at the correct stage in the *LKB1_S_KO* mice. Deeply staining elongated spermatid nuclei could be seen in the adluminal compartment of all *LKB1_S_KO* seminiferous tubules whereas in normal mice spermiation results in an absence of elongated spermatids in tubules in the two or three stages following spermiation. The relative absence of spermatozoa reaching the epididymis would suggest spermatids are never released and are instead phagocytosed by the sertoli cells. This is also supported by the observation of many deeply staining degenerating elongate spermatids around all observed tubule lumens. These resemble residual bodies (the excess spermatid cytoplasm that is retained by the sertoli cells upon normal spermatid release) but in the *LKB1_S_KO* mice usually at least one nucleus and flagellum is visible within them. They are often multinucleate suggesting the cytoplasm from adjacent spermatids has fused together. It is likely therefore, that a failure of spermiation accounts for the apparent disorganised appearance of the luminal region of the seminiferous epithelium due to degrading spermatids. It is possible that most spermatids are never released and instead degrade and fuse together, eventually to be phagocytosed by the sertoli cells.

Spermiation is recognised as a process requiring a series of co-ordinated steps that prepare the spermatids for release, up until the final disengagement. These include the translocation of germ cells through the seminiferous epithelium from the blood-testis barrier to the luminal edge; relocalisation and removal of residual cytoplasm; breaking down of the sertoli:germ cell adherens junctions, known as ectoplasmic specialisations; formation and removal of tubulobulbar complexes; and the final disengagement of the germ cells into the lumen [29], [32]. Each of these steps is thought vital for spermiation to proceed correctly and so it is possible that more than one of these processes is disrupted in *LKB1_S_KO* mice.

Some previous reports of mouse models showing a failure in spermiation have suggested that there is observed a defect in the breakdown of the ectoplasmic specialisations between the sertoli cells and spermatid heads. These include male mice lacking expression of the Sox8 transcription factor which is expressed by sertoli cells [33], and mice lacking expression of the endocytic receptor trafficking protein, EDH1 [34]. In the EDH1 study, the spermiation failure was accompanied by the presence of clumped spermatids and aggregated residual bodies within the seminiferous epithelium. It was proposed that EDH1 may play a role in the endocytosis and recycling of ectoplasmic specialisation components. In the current study, TEM analysis of ectoplasmic specialisations around retained spermatids in the *LKB1_S_KO* mice shows that these junctions are still present at stages when they should have already broken down. These could be identified by actin filament bundles adjacent to the spermatid heads. The apical ectoplasmic specialisations ES is an atypical actin-based adherens junction and is thought to share components and properties of adherens junctions, focal contacts and tight junctions [30], [35]. The mechanism by which these junctions are regulated is still not well understood. A number of peripheral protein kinases and phosphatases have been shown to associate with them, such as the tyrosine kinase c-Src, focal adhesion kinase and myotubularin related protein-2, a lipid/protein phosphatase [36], [37], [38]. The PAR3/PAR6 polarity complex has also been implicated in ectoplasmic specialisation restructuring and spermiation [39]. In addition, special germ cell cytoplasmic extensions known as tubulobulbar complexes have been suggested to play a role in the breakdown of the junctions [40]. Whether dysregulation of any of these protein complexes is involved in the phenotype of the *LKB1_S_KO* mouse model remains to be determined. LKB1 has previously been implicated in the regulation of tight junctions and adherens junctions [41], [42], [43] and it is therefore tempting to speculate that LKB1 may play a role in junction dynamics in testis. In the drosophila eye, loss of LKB1 was shown to cause an expansion of adherens junctions [43], whereas in the LKB1-deficient pancreas of mice, tight junctions and adherens junctions were absent [41].

Overall, there are a number of processes that may be disrupted in the *LKB1_S_KO* mice to cause the failure of spermiation. For example, the translocation of spermatids to the lumen is thought to be due to the association of the ectoplasmic specialisation with microtubule motor proteins within the sertoli cells which are then involved in transporting the junctions and therefore germ cell to the lumen [44]. LKB1 has previously been implicated in the regulation of microtubule dynamics via the MARK family of AMPK-related kinases [45], [46], which raises the possibility that decreased MARK activity in the *LKB1_S_KO* mice interferes with this process. It is also possible that there is a defect in the removal of residual cytoplasm from the spermatids. This could be a reason why spermatid heads and tails around the lumen often appear buried in large amounts of cytoplasm. If the cytoplasmic lobes are not released properly from the spermatids, they may become fused together with the spermatids still attached. Excess retained cytoplasm could also be an explanation for the detachment of the acrosomes from spermatid nuclei. In *LKB1_S_KO* mice acrosomal development appears to progress normally but then the acrosomes ‘peel off’ the nuclei of many late spermatids. A similar phenomenon of acrosome detachment has also been reported in mice that have a deletion of histone H1 variant, H1T2 [47]. It was suggested that in the case of H1T2 deletion this is due a defect in the elimination of residual cytoplasm during elongation. This pulls the outer membrane, with the acrosome attached, away from the spermatid head. This is similar to the situation in *LKB1_S_KO* mice where the detached acrosome usually stays attached to the outer membrane, with the ectoplasmic specialisation still visible linking it to the sertoli cells. It cannot be ruled out that problems in spermiogenesis may have occurred prior to the defect in spermiation. The sertoli cells may be able to detect that the spermatids are abnormal and therefore retain them for phagocytosis.

LKB1 has previously been described as a ‘master kinase’ with many different functions. It is therefore very possible that a number of downstream kinases and pathways are affected in *LKB1_S_KO* mouse testis. Of the 14 kinases shown previously to be substrates of LKB1, there have been few studies characterizing the tissue distribution of the proteins. So far, AMPKα1/α2 [48], BRSK1/2 [11], SNRK [12] and NUAK2 [49] proteins have been shown to be present in testis and our RT-PCR data has detected RNA for the remainder of the AMPK-related kinases in testis. The expression of some of those studied shows a similar temporal expression pattern to LKB1_S_ in early testis development, particularly AMPKα1. Comparison of the catalytic activities of AMPK and several of the AMPK-related kinases showed that their activities were all reduced in testis from *LKB1_S_KO* mice relative to wild-type. The greatest reductions in activity were seen for BRSK2, NUAK2 AMPKα2 and SNRK. It is interesting to note that the kinases with the greatest reductions in activity do not always show similar developmental expression profiles to LKB1_S_. For example, SNRK expression does not correlate with that of LKB1_S_ during testis development and yet its activity is reduced by more than 60% in *LKB1_S_KO* mice. These findings underscore the likelihood that functional kinase activity is not necessarily directly related to mRNA levels. Other factors, including protein stability and post-translational modification may play an important role in determining kinase activity *in vivo*. Due to there being a number of different cell types in testis it is hard to draw any firm conclusions as to the downstream substrates of LKB1_S_. For example, in wild-type mice some kinases may be expressed in a number of different cell types that normally express different relative amounts of LKB1_L_ and LKB1_S_. If the activity of one kinase was completely lost in developing spermatids due to the absence of LKB1_S_, it may still be highly expressed and active in another cell type where LKB1_L_ is still present, thus masking the loss in germ cells. Unfortunately, specific antibodies that could be used in cellular localisation studies were unavailable. A study looking at the activity of AMPK and individual AMPK-related kinases specifically in spermatids may be informative. Interestingly, in rats SNRK has previously been suggested to be testis-specific [12] making this a potential candidate in the infertility phenotype. In addition, AMPK has previously been implicated in the provision of lactate to germ cells, their main energy source, via the regulation of glucose and lactate transporters on the surface of sertoli cells [50], [51]. Whether these processes are disrupted in *LKB1_S_KO* mice is unknown.

Whilst this model unequivocally demonstrates thatLKB1 is an important regulator of male fertility, a limitation of the current model is that it is not clear whether the defects seen are a result of absence of LKB1_S_ expression, downregulation of LKB1_L_ expression, or a combination of both effects. Despite this, there is good evidence to suggest that the LKB1_S_ splice variant plays a significant role in spermatogenesis due to its high expression in spermatids relative to LKB1_L_.

In conclusion, the primary cause of the infertility in *LKB1_S_KO* mice is a defect in spermatid release from the seminiferous epithelium. A future aim is to identify the protein kinase target(s) downstream of LKB1 involved in spermiation. At present, many of the processes involved in spermiation are not well understood. Our finding that LKB1 plays a critical role in spermiation brings us a step closer to understanding the molecular mechanisms involved in this complex process, the regulation of which is a potential target for male contraceptives and infertility therapies.
